# Non-invasive High Frequency Median Nerve Stimulation Effectively Suppresses Olfactory Intensity Perception in Healthy Males

**DOI:** 10.3389/fnhum.2018.00533

**Published:** 2019-01-21

**Authors:** Ashim Maharjan, Mei Peng, Yusuf O. Cakmak

**Affiliations:** ^1^Department of Anatomy, School of Biomedical Sciences, University of Otago, Dunedin, New Zealand; ^2^Department of Food Science, University of Otago, Dunedin, New Zealand; ^3^Brain Health Research Centre, Division of Sciences, University of Otago, Dunedin, New Zealand; ^4^Medical Technologies Centre of Research Excellence, Auckland, New Zealand

**Keywords:** median nerve stimulation, near-infrared spectroscopy, orbitofrontal cortex, olfaction, labeled magnitude scale, non-invasive electrostimulation, nausea and vomiting

## Abstract

Median nerve stimulation (MNS) had been performed in the existing literature to alleviate symptoms of nausea and vomiting. The observed facilitative effects are thought to be mediated by the vagal pathways, particularly the vagus nerve (VN) brainstem nuclei of the dorsal motor nucleus of vagus and nucleus tractus solitarius (DMV-NTS). Sense of smell is one of the major sensory modalities for inducing vomiting and nausea as a primary defense against potentially harmful intake of material. This study aimed to test effects of non-invasive, high and low frequency MNS on human olfactory functioning, with supplementary exploration of the orbitofrontal cortex (OFC) using near-infrared spectroscopy (NIRS). Twenty healthy, male, adults performed supra-threshold odor intensity tests (labeled magnitude scale, LMS) for four food-related odorant samples (presented in three different concentrations) before and after receiving high-, low frequency MNS and placebo (no stimulation), while cortical activities in the OFC was monitored by the NIRS. Data of the NIRS and LMS test of separate stimulation parameters were statistically analyzed using mixed-model analysis of variance (ANOVA). Only the high frequency MNS showed effects for suppressing the intensity perception of the moderate concentration of Amyl Acetate (p:0.042) and strong concentration of Isovaleric Acid (p:0.004) and 1-Octen-3-ol (p:0.006). These behavioral changes were coupled with significant changes in the NIRS recordings of the left (p:0.000) and right (p:0.003) hemispheric orbitofrontal cortices. This is the first study that applied non-invasive, high frequency MNS to suppress the supra-threshold odor ratings of specific concentrations of odors. The vagal networks are potential relays of MNS to influence OFC. Results from the current article implore further research into non-invasive, high frequency MNS in the investigation of its modulatory effects on olfactory function, given its potential to be used for ameliorating nausea and malnutrition associated with various health conditions.

## Introduction

Sense of smell, in combination with vision and taste, are essential contributors to inducing nausea or vomiting as the primary defense against dangerous or contaminated food (Andrews, 1992). The link between odor and nausea even dates back to the Roman empire where invaders were thought to give off a nauseating odor, caused by the use of rancid butter as hair ointment (Classen et al., 1994). Nausea and vomiting are one of the most frequently reported symptoms in clinical settings due to the wide range of conditions including medications, motion sickness, infections, hormonal disorders, pregnancy, central nervous system disorders, psychiatric disorders, anesthesia, and cardiovascular dysfunctions (Andrews, 1992; Babic and Browning, 2014; Lee and Fan, 2015). Furthermore, nausea and vomiting often coincide with a loss of appetite. For those who suffer from this cluster of symptoms for a long term, such as patients who undergo chemotherapy treatment, their quality of life can be substantially affected (Guerdoux-Ninot et al., 2016). Despite its high prevalence and numerous causes, the treatment options for nausea with medication are not highly effective and can also have side effects (Lee and Fan, 2015). Median nerve stimulation (MNS) is one of the alternative approaches that are being explored in the current literature to reduce nausea and vomiting.

MNS was initially performed using manual acupuncture on the median nerve (MN) [a traditional Chinese method using Neiguan acupuncture point (pericardium meridian of acupuncture point 6; PC-6)]. Over the past couple of decades, MNS has advanced from manual acupuncture techniques (Napadow et al., 2009; Bai et al., 2010; Lee and Fan, 2015) to electroacupuncture (EA) which is a modification of the acupuncture technique using electrical current (Spiegel et al., 1999; Chen et al., 2003; Tatewaki et al., 2005; Lee and Fan, 2015). A popular form of MNS in the current literature is the use of transcutaneous electrical nerve stimulation (TENS; Zatorre et al., 1992; Urasaki et al., 1998; Ferretti et al., 2007; Lee and Fan, 2015). In the current literature, MNS is used to treat gastrointestinal diseases (Diehl, 1999; Wang et al., 2000; Chang et al., 2001; Takahashi, 2006; Xu et al., 2006) and alleviating symptoms of nausea and vomiting in clinical settings that includes post-surgery/chemotherapy, pregnancy or motion sickness (Xu et al., 2006; Dhond et al., 2008; Ren et al., 2010; Zotelli et al., 2014).

In the existing literature, MNS has been used to alleviate symptoms of nausea and vomiting, effects that are thought to occur through the vagal pathways (Chen et al., 2003; Tada et al., 2003; Tatewaki et al., 2005; Xu et al., 2006; Bai et al., 2010; Ren et al., 2010; Takahashi, 2011; Zotelli et al., 2014; Lee and Fan, 2015). The use of low frequency (10–25 Hz) MNS has also demonstrated a positive control of gastric mobility *via* increased gastric emptying and the ability to regulate gastric dysthymia (Chen et al., 2003; Tatewaki et al., 2005; Xu et al., 2006). As the vagus nerve (VN) is recognized as the sole contributor to the control of gastric acid secretion and mobility, this tantalizes VN and MNS interactions. In both animal models and human studies, MNS has displayed the ability to modulate the VN (Ouyang et al., 2002; Chen et al., 2003; Xu et al., 2006; Takahashi, 2011). In a previous article by Ouyang et al. (2002), low frequency MNS accelerated gastric emptying of liquids and improved gastric slow-wave rhythmicity. Modulation of gastric function using MNS was also absent after vagotomy in animal models (Noguchi and Hayashi, 1996; Xu et al., 2006). This supports the notion that the potential pathway of MNS on gastric functions are through the dorsal motor nucleus of vagus-Nucleus Tractus Solitarius (DMV-NTS), both of which are VN brainstem nuclei (Ruggiero et al., 1998; Cakmak, 2006; Imai et al., 2008).

In a previous article by our group (Maharjan et al., 2018), non-invasive and high frequency auricular VN electrostimulation improved olfactory performance in the supra-threshold test. However, to date, the MNS’ potential effects on olfactory function *via* its interactions with vagal nerve networks has not been explored yet. Therefore, this study aimed to observe the effects of non-invasive MNS on olfactory sensory function using a labeled magnitude scale (LMS) test in healthy, male, adults under high and low frequencies of electrostimulation with supplementary exploration of the orbitofrontal cortex (OFC) using near-infrared spectroscopy (NIRS).

## Methods

### Participants

Twenty Caucasian-male, healthy, non-smokers (age range = 22–32 years, mean: 25.05 years, standard deviation: 2.36 years) participated in the current study. Prior to the study, each participant was asked not to consume any food or non-water beverages for 2 h prior to the experiments. They were also instructed to refrain from applying any fragrance product/s on the day of the experiment. All participants gave an informed, written consent to participate in the experiment, in accordance with the Declaration of Helsinki and met the exclusion criteria set for the experiment (non-smoker, in a healthy condition and were of NZ-European descent). The experiment was approved by the Otago Human Participants Ethics Committee (Reference: H16/148) and registered to the Australian New Zealand clinical trials registry (ANZCTR; registration ID: ACTRN12617000034336, Clinical trial name: MODOLF).

### Labeled Magnitude Scale (LMS) Test

The LMS test is a widely-used psychophysical scaling method for quantifying intensity perception of sensory stimuli (Green et al., 1993, 1996). The LMS is composed of seven verbal labels arranged according to the geometric means of their rated magnitude which ranges from 0 to 100 (“no sensation”-0, “barely detectable”-1.4, “weak”-6.1, “moderate”-17.2, “strong”-35.4, “very strong”-53.3, “strongest imaginable”-100). Following previous protocols (Green et al., 1993, 1996; Kalva et al., 2014), the current study tested responses to four different odors—two “pleasant” odors [Citral (CAS number: 5392-40-5; purity: 99%, Sigma-Aldrich, St. Louis, MO, USA)—commonly described as citrus and Amyl Acetate (CAS number: 628-63-7; purity: 99%, Sigma-Aldrich, St. Louis, MO, USA)—commonly described as banana] and two “unpleasant” odors [Isovaleric Acid (CAS number: 503-74-2; purity: 99%, Sigma-Aldrich, St. Louis, MO, USA)—commonly described as cheese and 1-Octen-3-ol (CAS number: 3391-86-4; purity: 99%, Sigma-Aldrich, St. Louis, MO, USA)—commonly described as mushroom]—with three different concentrations for each odor. Specifically, these odors included Citral (2 ppm, 20 ppm, 200 ppm), 1-Octen-3-ol (3 ppm, 18 ppm, 108 ppm), Amyl Acetate (1.5 ppm, 15 ppm, 150 ppm) and Isovaleric acid (4 ppm, 28 ppm, 196 ppm). All odors were diluted in distilled water. The selection of these concentrations underwent four stages of bench-top testing (*n* = 6–10) which were performed to differentiate three concentrations (weak, moderate and strong) for each odorant. Participants used the LMS scale to rate each concentration of each odor in the bench-top testing stages. After four stages of bench-top testing, the use of specific concentrations for each odor matched the ratings (weak, moderate and strong) in the LMS to a satisfactory level. All results of bench-top testing are provided in Supplementary Table S1.

An additional screening criterion used in the initial studies of the LMS test (Green et al., 1996) was also included to ensure that each participant could differentiate weak, moderate and strong concentrations of each odor (Citral, Amyl Acetate, Isovaleric Acid, 1-Octen-3-ol). In this instance, we used Ethyl-butyrate (CAS number: 105-54-4; purity: 99%, Sigma-Aldrich, St. Louis, MO, USA) using 1.25 ppm as weak concentration, 13.69 ppm as moderate concentration and 150 ppm as strong concentration. These concentrations of Ethyl-butyrate also underwent four stages of bench-top testing (Supplementary Table S1). This odor was chosen as it was distinct from the odors used in the testing phase of the current study. Before the start of the pre-stimulation and post-stimulation LMS, each participant was given a separate set of glass lidded bottles with an example of a weak and strong concentration of an independent odor distinct to the testing odors (Ethyl-butyrate: weak-1.25 ppm and strong-150 ppm). Each concentration of all four odors was presented in a 240-ml glass lidded bottles. To sample an odorant, the subject was asked to place the glass bottle approximately 2 cm away from the nose and inhale within a 2–3 s window. Separate sets of randomized numeral labeled bottles were used for pre-stimulation and post-stimulation LMS. Each concentration was presented once (in both pre-stimulation LMS and post-stimulation LMS), in a randomized order that was counterbalanced for each stage (pre- and post-stimulation LMS) across all participants. 30 s inter-stimulus interval was present between the presentation of each odor. The participants’ response on the LMS was measured using the software “Compusense” (Compusense Cloud, Version 8.8.6766.17069, Compusense Inc., Guelph, ON, Canada).

### Application of Median Nerve Stimulation (MNS)

**Figure 1:**
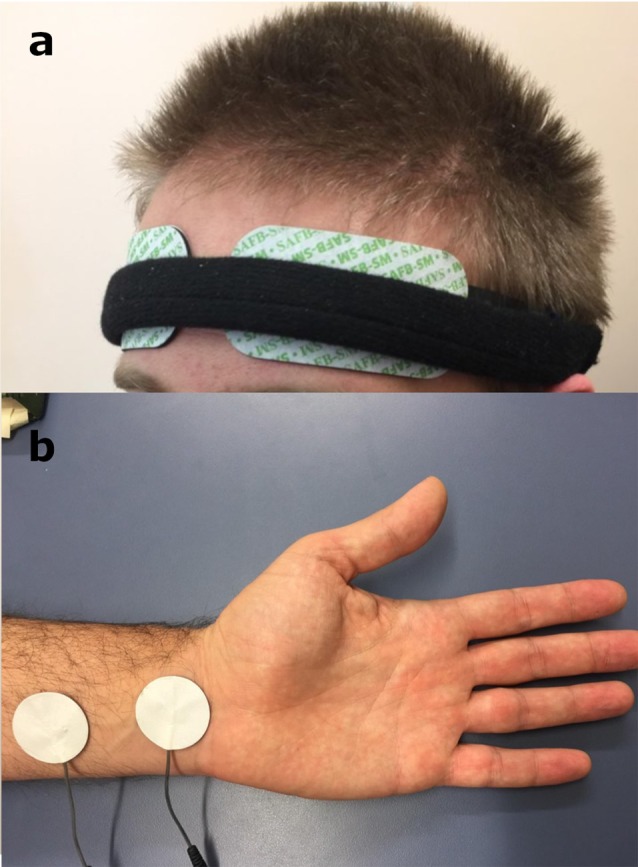
**(a)** Near-infrared spectroscopy (NIRS) electrodes set up on the forehead of the participant. **(b)** The location of the non-invasive median nerve stimulation (MNS) using the pair of disposable rubber electrode-pads on the MN region of the left forearm. Written informed consent was obtained from the participant for the publication of this image (Supplementary Figure S1).

Non-invasive MNS was applied to the participant using “TENS ECO-2” (SCHWA-MEDICO, 35630 Ehringshausen, Germany) by the experimenter, using TENS for all three different parameters—high frequency MNS (80 Hz), low frequency MNS (10 Hz) and placebo (no stimulation but the electrodes were still attached). TENS in the current experiment was performed with the use of two pairs of disposable rubber electrode-pads, placed on the MN region of the left forearm, with the cathode being 3 cm proximal to the anode (Figure 1b), similar to previous studies using MNS with TENS (Urasaki et al., 1998; Ferretti et al., 2007). The TENS intensity was adjusted to obtain a tingling sensation over the MN territory of the palm and visible flexion response of index and/or middle finger (and/or thumb). This indicated the stimulation of the sensory and motor fibers of the MN in each stimulation session. In addition, the stimulation intensity was also kept to a comfortable range for the participant to ensure there was no perception of pain. The strength of the MNS (amplitude) was between 8 mA and 15 mA and the pulse bandwidth was 180 μS width bipolar square waveform.

### Procedure

**Figure 2:**
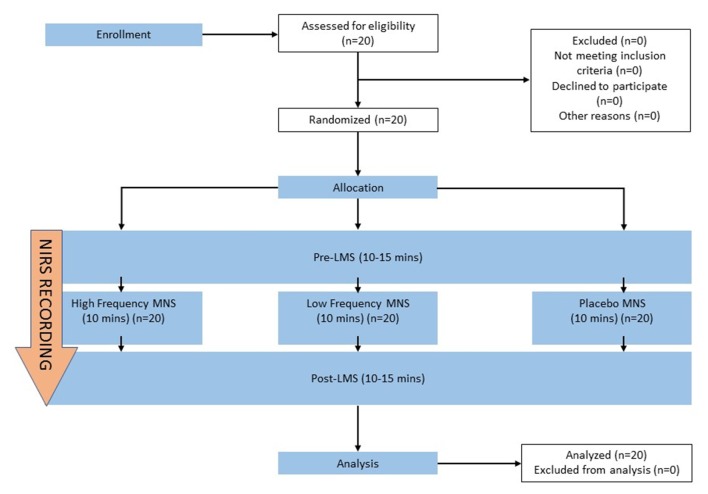
Schematic sequence of each stage of the current study. The sequence of the experimental stages includes pre-stimulation labeled magnitude scale (Pre-LMS), allocated stimulation parameter (high frequency MNS, low frequency MNS, placebo) and post-stimulation (Post-LMS). A brief 5-min introduction, prior to the experiment comprised of obtaining participant’s consent and device set up. MNS, LMS, NIRS recordings from both hemispheres of the orbitofrontal cortex (OFC) were recorded simultaneously across all stages of the experiment (Pre-LMS, allocated stimulation parameter, Post-LMS) for all participants.

Each participant was given a brief introduction to all the stages of the experiment and were instructed to attend three, 30–45-min sessions with at least 24-h apart. The experimental room was an isolated environment with air conditioning, allowing for a consistent temperature (23 ± 1°C) in the absence of any olfactory or visual stimuli representing food or distractions. The participants were instructed not to sniff during the olfactory tests to eliminate the potential effects of sniffing itself during the olfactory tests and NIRS recordings. The participant was seated directly opposite to the experimenter. A consent form and exclusion criteria sheet were signed to qualify the participants for the study. A brief explanation of the full experimental process and the olfactory test (LMS) were given to the participant. Pre-stimulation, LMS was performed for 10–15 min, followed directly by the allocated stimulation parameter for 10 min (high frequency MNS, low frequency MNS or placebo) that was delegated randomly for each participant. Lastly, post-stimulation, LMS (10–15 min) was performed directly after the allocated stimulation parameter (Figure 2). This study followed a double-blind design.

Figure 2 details the experimental design of the current study. A within-participant design was enforced. In each session, participants were randomly assigned to one of the three experimental conditions: high frequency MNS, low frequency MNS or placebo. The order of experimental conditions was counterbalanced across the participants. In addition to the exclusion criteria put in place to ensure that all participants were in healthy condition for the experiment, University of Pennsylvania Smell Identification Test (UPSIT) odor identification test (OIT; Sensonics International, Haddon Heights, NJ, USA) and odor discrimination/memory test (ODMT; Sensonics International, Haddon Heights, NJ, USA) were performed by each participant, after the placebo session. The results from these olfactory tests were compared to that of previous studies (Doty et al., 1984, 1995; Choudhury et al., 2003) to ensure that all participants met the standard for normative, healthy response in healthy, adult, male subjects.

### Near-Infrared Spectroscopy (NIRS)

NIRS (COVIDIEN INVOS OXIMETER, Model 5100C-PA, Mansfield, MA, USA) was performed in the current study to measure participants’ activity from the OFC. This INVOS (*in vivo* Optical Spectroscopy)-NIRS model is a clinically validated and FDA approved device, used in over 600 peer-reviewed articles and three randomized controlled trials (Harrer et al., 2010; Edmonds et al., 2016, 2017). The setup of this device is shown in Figure 1a. In the existing literature, several studies have demonstrated the efficiency of the NIRS in monitoring the activation of the OFC under olfactory stimulus (Hongo et al., 1995; Cho et al., 1998; Edmonds et al., 1998; Ishimaru et al., 2004; Harada et al., 2006; Kobayashi et al., 2012). With the aid of fMRI localization, olfactory expression was understood to be present in the lateral and anterior orbito-frontal gyri of the frontal lobe. With this knowledge, several studies have recorded from the orbito-frontal region using NIRS techniques and found that oxygenated hemoglobin (HbO_2_) concentration increased only over the orbito-frontal region during olfactory stimulation (Ishimaru et al., 2004; Harada et al., 2006; Kobayashi et al., 2012).

INVOS-NIRS facilitates non-invasive method of monitoring regional tissue oxygenation (oxy- and deoxyhemoglobin) by using the modified Beer-Lambert law for light attenuation changes through the illuminated tissue (relating to optical density to chromophore concentration). INVOS-NIRS measures the red pigment of hemoglobin molecules within the red blood cells which is understood to have the highest light absorption of the wavelengths (730 and 810 nm; Edmonds et al., 2004; Covidien, 2013). The INVOS-NIRS system sensors used two near-infrared light sources at two different wavelengths (730 and 810 nm) and two photodiode detectors at a distance to observe the oxygen-hemoglobin saturation of the tissues beneath the sensors. The sensor’s light emitting diode sends light to either a proximal or distal detector in a parabolic path, which allows the processing of separate data of shallow and deep optical signals. With the use of an algorithm of subtraction of the short vs. long travel distance of the light, INVOS-NIRS enables high spatial resolution (effective localization of the area of measurement) and enables the elimination of skin and scalp data (Hongo et al., 1995; Edmonds et al., 2004; Covidien, 2013). With the wavelengths of 730–810 nm, it is worth mentioning that melanin and water also behave as chromophores, but fortunately, INVOS-NIRS has shown in the existing literature that recordings have been unaffected by normal skin pigmentation in adults (Misra et al., 1998; Damian and Schlosser, 2007).

By measuring regional hemoglobin oxygen saturation (rSO_2_), the INVOS-NIRS system provides absolute, real time data accuracy from the cortex with the use of multi-sensors within the same sensor and is empirically validated in human subjects (Hongo et al., 1995; Cho et al., 1998; Edmonds et al., 1998, 2004; Roberts et al., 1998; Higami et al., 1999; Singer et al., 1999; Yao et al., 2001; Alexander et al., 2002; Iglesias et al., 2003). rSO_2_ reflects the balance between cerebral oxygen supply and demand, measuring the continuous local brain oxygen balance in a non-invasive approach (Cho et al., 1998; Edmonds et al., 1998, 2004; Blas et al., 1999; Janelle et al., 2002; Prabhune et al., 2002; Casati et al., 2005; Harada et al., 2006).

Unlike other systems that only assesses venous or arterial blood, the current study used the clinically validated and FDA approved INVOS-NIRS, which measures a 3:1 ratio of venous and arterial blood. This algorithm was calibrated using blood samples with the assumption that 25% of the blood within the sample volume being arterial while 75% of the blood within the sample volume was venous (Edmonds et al., 2004) This represented a venous-blood saturation (venous-weighted percent of rSO_2_-VWrSO_2_%) which can be used to provide real-time data about the balance (or imbalance) of oxygen supply/demand. Thus, this reflects venous oxygen reserve (VOR; oxygen remaining from extraction of associated tissues and vital organs) providing accurate measurement of site-specific tissue oxygenation (Blas et al., 1999; Janelle et al., 2002; Prabhune et al., 2002; Casati et al., 2005). In the current literature, reduction in VOR is considered a warning sign of developing pathology or deteriorating patient condition with reports of 20%–25% from baseline or rSO_2_ of 40–50 considered to be cause of concern in association with neurologic dysfunction and other adverse outcomes (Hongo et al., 1995; Cho et al., 1998; Edmonds et al., 1998, 2004; Roberts et al., 1998; Higami et al., 1999; Singer et al., 1999; Yao et al., 2001; Alexander et al., 2002; Iglesias et al., 2003; Murkin et al., 2007; Ballard et al., 2012; Vretzakis et al., 2013).

The current study also used disposable electrodes for each participant to ensure highest hygiene and data quality, in addition to using a rigid head band to stabilize the electrodes and the cables that are attached to the electrodes (Figure 1a). We measured from the forehead region (Figure 1a) to avoid issues regarding hair or hair follicles that can produce excessive photon scattering in INVOS-NIRS resulting in artificial low rSO_2_ (Oriheula-Espina et al., 2010). Furthermore, this forehead region is the routine zone of cerebral oximetry monitoring by INVOS-NIRS and has FDA validated approval for the use of this device for cerebral monitoring (Prabhune et al., 2002; Casati et al., 2005; Edmonds et al., 2017). INVOS-NIRS system also provides a reliable signal strength index (SSI) for stable recordings with each channel showing a 5-unit bar scale system. Any signal over 1 bar indicates a stable recording to generate an accurate VWrSO_2_ (%) recording [Chapter 6.4.16 and 11.7.4 in the INVOS-NIRS 5100c manual (Covidien, 2013)]. In the current study, we ensured that the SSI bar was at the highest signal (5/5 SSI) throughout all of the recordings. We only enrolled healthy participants in the current study to eliminate potential pathological artifacts (cranial bone anomalies, frontal sinus inflammation or dyshemoglobinemia; Gopinath et al., 1995; de Letter et al., 1998; Madsen et al., 2000; McRobb et al., 2011) as well as maintaining a consistent room temperature and position of participant throughout the experiment to avoid any potential anomalies with the INVOS-NIRS measurements. With the INVOS-NIRS recordings, each section of the experiment (Figure 2), was mapped to ensure that each stage of the experiment had an average (mean) recording of VWrSO_2_ (representing VOR) for each participant from the left and the right hemispheres of the OFC.

### Data Analysis

Data from the NIRS device was transferred to the INVOS software which accommodated data presentation. We used average (mean) recordings of VWrSO_2_ (%) data of specific time periods marked for each segment of each session which was in line with the previous research (Cho et al., 1998; Murkin et al., 2007). To insure that the baseline activity was consistent across all three different stimulation parameters for LMS and LMS-NIRS data, repeated-measures analysis of variance (ANOVA) was also applied to the data that was obtained at the pre-stimulation stage (Table 1). Mixed-model ANOVA was applied to data obtained from each stimulation parameters (i.e., high frequency MNS, low frequency MNS and placebo) for assessing VWrSO_2_ (%) changes in separate hemispheric OFC (independent variable: left and right) across stages of the experiment (repeated-measures variable: pre-stimulation LMS, stimulation parameter and post-stimulation LMS). For the LMS data, mixed-model ANOVA (repeated-measures variable: pre-stimulation LMS, post-stimulation LMS; independent variables: stimulation, odor, concentration) were performed to assess differences between the scores before and after stimulation for all three parameters (high frequency MNS, low frequency MNS, and placebo). *Post hoc* test, based on simple effects tests with Bonferroni correction, was applied to understand any significance at *p*-value of 0.05. All the analyses were performed using SPSS (IBM SPSS Statistics, Ver. 20, St Leonards, NSW, Australia).

**Table 1:** Pre-stimulation results of the repeated-measures analysis of variance (ANOVA) that assessed the potential difference (if any) of the labeled magnitude scale (LMS) test and the near-infrared spectroscopy (NIRS; VWrSO_2_%) data in all stimulation parameters [high frequency median nerve stimulation (MNS), low frequency MNS and placebo].

Olfactory test and NIRS recordings (h = hemisphere)	Stimulation parameter (H-High, L-Low, P-Placebo)	Mean	SD	*F*-Value	*p*-value
Pre-S LMS Mod AA	H	16.2225	12.8618	0.910	0.411
	L	13.4375	9.7741		
	P	17.3500	12.3399		
Pre-S LMS Str VA	H	22.8375	16.9355	0.015	0.985
	L	22.2125	20.4004		
	P	22.7275	22.4596		
Pre-S LMS Str Octen	H	29.9650	23.4717	0.821	0.448
	L	27.8775	21.0517		
	P	32.3925	21.2645		
Left-h VWrSO_2_ at Pre-S LMS	H	70.9675	9.3354	2.259	0.118
	L	70.8867	8.8145		
	P	72.7027	9.5207		
Right-h VWrSO_2_ at Pre-S LMS	H	72.9550	7.5862	1.049	0.360
	L	71.2606	8.0289		
	P	72.7104	9.7302		

*SD, standard deviation; LMS score ranges, 0–100; NIRS, 0–100%; H, high frequency MNS; L, low frequency MNS; P, placebo condition; left-h, left hemisphere; right-h, right hemisphere; mod AA, moderate concentration of Amyl Acetate; Str VA, Strong concentration of Isovaleric Acid; Str Octen, Strong concentration of 1-Octen-3-ol; Pre-S, Pre-Stimulation*.

## Results

### Pre-screening Olfactory Test Results—Odor Identification Test (OIT) and Odor Discrimination/Memory Tests (ODMT)

Half of the 20 participants displayed values of Mild Microsmia (30–33 out of 40 in OIT) while the other half of the participants displayed values of Normosmia (34–40 out of 40 in OIT). None of the participants in the current study displayed total anosmia which was the exclusion criteria for the current study and therefore, all participants presented olfactory performances in OIT to a standard held in line with the existing olfactory research (Doty et al., 1984, 1995). All participants scored between 8 and 12 out of 12 in the ODMT which corresponds to healthy ranges, in line with existing literature (Doty et al., 1984, 1995; Choudhury et al., 2003; Doty, 2003). In this context, all participants were qualified to take part in the current study.

### Labeled Magnitude Scale (LMS) Test Results

**Figure 3:**
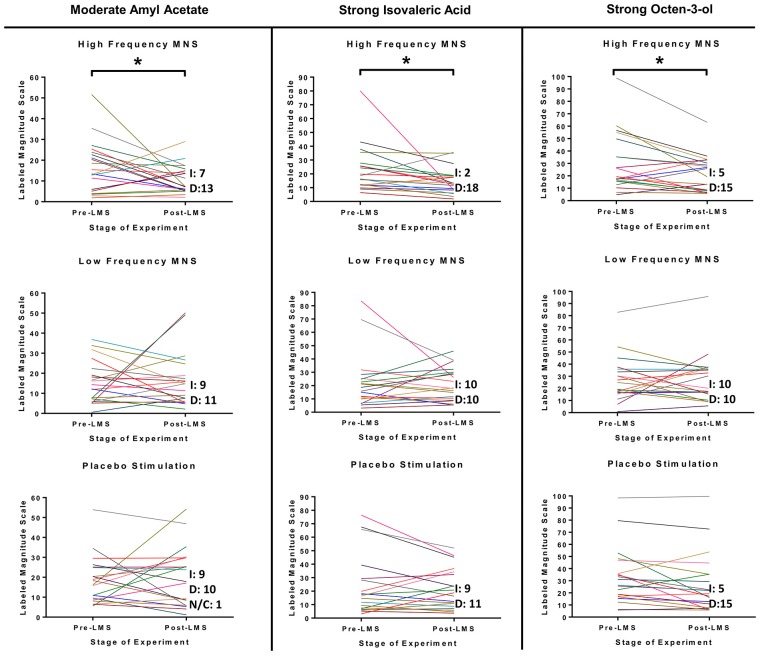
LMS test results for moderate concentration of Amyl Acetate, strong concentration of Isovaleric Acid and strong concentration of 1-Octen-3-ol from each participant before and after each stimulation parameter (high frequency MNS, low frequency MNS and placebo). Each participant’s scores, before (Pre-S LMS) and after (Post-S LMS) each of the stimulation parameters (high frequency MNS, low frequency MNS and placebo), for the LMS (LMS scores range = 0–100). Each colored line represents one case (total cases = 20). Letter “I” corresponds to cases that increased LMS test score in Post-S LMS in comparison to Pre-S LMS, letter “D” corresponds to cases that decreased LMS test score in Post-S LMS in comparison to Pre-S LMS and letters “N/C” corresponds to no change in LMS test score in Post-S LMS in comparison to Pre-S LMS. *Statistically significant (*p* < 0.05).

Inspection of changes in LMS scores of all participants (*n* = 20) between the pre-stimulation and post-stimulation stages in all three stimulation parameters (high frequency MNS, low frequency MNS and placebo) suggested significant differences for only the high frequency MNS (Figure 3). Specifically, significant post-stimulation declines were evident for moderate concentration of Amyl Acetate (*post hoc* pair wise analysis with Bonferroni, pre-stimulation-post-stimulation, p:0.042, *Partial Eta Squared*: 0.006, low size effect), strong concentration of Isovaleric Acid (*post hoc* pairwise analysis with Bonferroni, pre-stimulation-post-stimulation, p:0.004, *Partial Eta Squared*: 0.012, low size effect) and strong concentration of 1-Octen-3-ol (*post hoc* pairwise analysis with Bonferroni, pre-stimulation-post-stimulation, p:0.006, *Partial Eta Squared*: 0.011, low size effect). Ratings for moderate concentration of Amyl Acetate, strong concentration of Isovaleric Acid and strong concentration of 1-Octen-3-ol were reduced in the post-stimulation LMS stage in comparison to the pre-stimulation LMS stage after high frequency MNS. Notably, there were no significant differences between the pre-stimulation and post-stimulation LMS stages in all concentrations of Citral, weak and strong concentrations of Amyl Acetate, and weak and moderate concentrations of Isovaleric Acid and 1-Octen-3-ol after high frequency MNS. Under low frequency MNS and placebo conditions, no significant changes were observed for any odor in any of the different concentrations (Supplementary Figures S2–S6).

### All Stage NIRS Data Analysis

**Figure 4:**
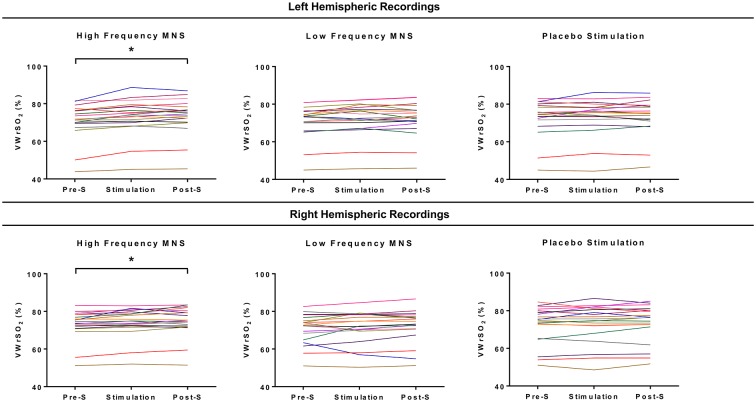
Each of the participant’s recordings for all three stages of the experiment (pre-stimulation LMS, stimulation parameter, post-stimulation LMS) for each stimulation parameter (high frequency MNS, low frequency MNS and placebo) from both the left and right hemispheres of the OFCs, measuring venous oxygen reserve (VWrSO_2_%) using NIRS. Each colored line represents one case (total cases = 20). *Statistically significant (*p* < 0.05).

Individual results from each participant’s (*n* = 20) recording of VWrSO_2_ (%) from the left and right hemispheres of the OFC is displayed in Figure 4. Significant differences across the three stages (pre-stimulation LMS, MNS/placebo, post-stimulation LMS) of VWrSO_2_ (%) recordings were only present in the high frequency MNS parameter in both hemispheres of the OFC (left hemisphere: p:0.000, *Partial Eta Squared*: 0.463-large effect size; right hemisphere: p:0.003, *Partial Eta Squared*: 0.270-large effect size). *Post hoc* pairwise analysis with Bonferroni correction indicated that in the left and right hemispheres of the OFC, there were significant differences in VWrSO_2_ (%) recordings between the pre-stimulation and stimulation stages (left hemisphere p:0.000; right hemisphere p:0.016) and pre-stimulation and post-stimulation stages (left hemisphere p:0.000; right hemisphere p:0.002). There were no significant differences between the left and the right hemispheric recordings of the OFC (p:0.592). The NIRS recordings of VWrSO_2_ (%) increased in the stimulation and post-stimulation stages in comparison to the pre-stimulation stage in both hemispheres.

### Pre-stimulation, Intergroup Differences of Labeled Magnitude Scale (LMS) and Respective Near-Infrared Spectroscopy (NIRS) Recordings From Orbitofrontal Cortices (OFC) Convergent and Divergent Co-transmission

Table 1 displays the results of the repeated-measures ANOVA that assessed the potential differences (if any were present) in the significant results from the LMS test (pre-stimulation vs. post-stimulation) and the NIRS recordings (VWrSO_2_%) in all of the three different stimulation parameters (high frequency MNS, low frequency MNS, placebo) at the pre-stimulation stage of testing. As indicated in Table 1, there is no significant differences in the pre-stimulation stage for all significant results of the LMS test (moderate concentration of Amyl Acetate, strong concentration of Isovaleric Acid and strong concentration of 1-Octen-3-ol) and NIRS (VWrSO_2_%) recordings under any of the stimulation parameters for both hemispheres of the OFC.

## Discussion

**Figure 5:**
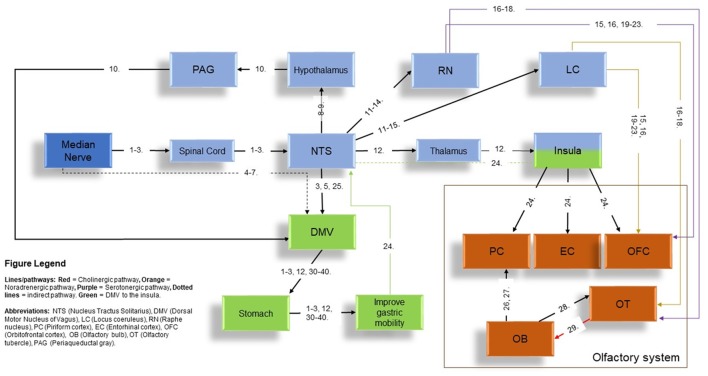
The potential pathways to the olfactory network *via* MNS. Figure legend in the image indicates all abbreviations and the type of pathways (represented by different colors) present in the current diagram. Structures in color “blue” refer to the indirect stimulation of the vagus nerve (VN) pathway using MNS to the olfactory network while structures in color “green” refer to the stimulation of the Nucleus Tractus Solitarius-dorsal motor nucleus of vagus (NTS-DMV; VN brainstem nucleus) and subsequent interactions with the insula to the olfactory network *via* MNS. Orange structures represent the structures of the main olfactory system. Numbers in the figures represent references provided in a separate document in Supplementary Table S2. Abbreviations of structures are given in the Figure Legend on the bottom-left corner of the diagram.

Studies of MNS in the existing literature have primarily focused on the cortical activation of primary (S-I) and secondary (S-II) somatosensory cortex (Spiegel et al., 1999; Nihashi et al., 2005; Napadow et al., 2009; Zhang et al., 2012; Zotelli et al., 2014), improvement of gastric function (Ouyang et al., 2002; Chen et al., 2003; Tatewaki et al., 2005; Xu et al., 2006; Takahashi, 2011) and alleviating symptoms associated with nausea and vomiting (Tatewaki et al., 2005; Xu et al., 2006; Bai et al., 2010; Takahashi, 2011; Zhang et al., 2012; Zotelli et al., 2014; Lee and Fan, 2015). As the VN is the key contributor towards gastric acid secretion and mobility, the modulation of gastric function by MNS addressed a potential interaction between MNS and VN. This interaction is supported in animal models and human studies (Ouyang et al., 2002; Chen et al., 2003; Xu et al., 2006; Takahashi, 2011). In the current literature, low frequency MNS has indicated several functions in regards to gastric mobility that includes accelerated gastric emptying of liquids and improved gastric slow-wave rhythmicity (Ouyang et al., 2002), effects absent after vagotomy (Noguchi and Hayashi, 1996; Xu et al., 2006). In a previous article by our group (Maharjan et al., 2018), non-invasive and high frequency auricular VN electrostimulation improved olfactory performance in the supra-threshold test. This indicated that high frequency, VN stimulation (VNS) can modulate olfactory function with its neural connections (Figure 5). In the context of modulation of olfactory function with high frequency VNS in our previous study and the existing literature on MNS’ effects on changes in gastric mobility and secretion [which is also driven by the DMV and viscerosensory VN-NTS and subsequently to the insula-olfactory networks (Figure 5)] (Ruggiero et al., 1998; Cakmak, 2006; Imai et al., 2008; Mayer, 2011), we conceptualized that high frequency MNS may have a modulatory effect on olfactory function.

On the other hand, to date, MNS studies primarily used low frequencies (1.5–25 Hz). MNS with low frequencies (1.5–4.0 Hz) showed maximal activation of the S-I and S-II in animals models (Gyngell et al., 1996) and humans studies (Ibáñez et al., 1995) while the use of 5 Hz resulted in an absence of activation in the S-I and the use of 15–30 Hz only had effects on some of the participants in the enrolled study (Puce et al., 1995). MNS using 0.5–1 Hz in conjunction with fMRI techniques in human subjects have found increased default network interconnectivity, greater connectivity between sensorimotor networks (Dhond et al., 2008) and increased activation of posterior insula, hypothalamus and cerebellum (Bai et al., 2010). Furthermore, low frequencies MNS of 10–25 Hz have been used on the modulation of gastric function (Noguchi and Hayashi, 1996; Ouyang et al., 2002; Xu et al., 2006; Imai et al., 2008). Low frequencies (1–25 Hz) have also been performed to alleviate symptoms of nausea and vomiting in animal models (Ouyang et al., 2002; Chen et al., 2003) and human studies (Xu et al., 2006; Bai et al., 2010). The use of MNS in higher frequencies (50 Hz) was only explored in a single study and resulted in reduced activation of the S-I in comparison to the use of lower frequencies of MNS (Davis et al., 1995). In the current study, the use of low frequency MNS (10 Hz) did not show any significant changes in the supra-threshold odor rating in the LMS test. This indicates that it is possible that the suppression of nausea and vomiting with MNS could be acting through different cortical structures that excludes the OFC and potentially separate mechanisms from the sense of smell for alleviating symptoms of nausea and vomiting. This requires further exploration in future research, coupled with the investigation of high frequency MNS in the alleviation of symptoms associated with nausea and vomiting as it could have stronger effects in comparison to low frequency MNS.

Results of the present study demonstrated that non-invasive high frequency MNS can reduce olfactory intensity ratings of the moderate concentration of Amyl Acetate, strong concentration of Isovaleric Acid and 1-Octen-3-ol in healthy participants. In contrast, non-invasive low frequency MNS and placebo groups did not show any effects on modulating olfactory sensory ratings in the LMS test. These results were also supported by statistically significant increase of VWrSO_2_ (%) in the NIRS recordings in both hemispheres of the OFC only under high frequency MNS but not in the low frequency stimulation or placebo conditions. This is the first study that indicates that MNS under high frequency stimulation can suppress odor intensity ratings of specific concentrations of several odors in combination with the increased and simultaneous activation in the OFCs in both hemispheres. The previous study by our group (Maharjan et al., 2018) demonstrated that non-invasive high frequency (80 Hz) electrostimulation of the auricular VN can improve the performance of supra-threshold test in healthy, adult male participants. This improvement in supra-threshold olfactory function was also supported by a significant improvement of VWrSO_2_ (%) in the NIRS recordings only in the right hemisphere (Maharjan et al., 2018). In the present study, high frequency electrostimulation (80 Hz) of the MN suppressed the supra-threshold function of odor intensity ratings in the healthy volunteers with the bilateral improvement of VWrSO_2_ (%) in the NIRS recordings in both hemispheres. Both studies’ outcomes underlined the significance of high frequency (80 Hz) electrostimulation on modulating the olfactory function and also indicated the potential contribution of the OFC that accompanies the significant results from the supra-threshold olfactory tests in both studies with VWrSO_2_ (%) recordings.

Although both electrostimulation techniques were capable of modulating olfactory supra-threshold function in healthy humans, use of 80 Hz electrostimulation on two different nerves modulated the supra-threshold olfactory function in opposite directions. In this context, it is worth to note the lateralization of the OFC for olfactory function. The right OFC, along with the right piriform cortex, is associated with increased activation during higher-order processing of smell sensation in comparison to the left hemispheric counterparts (Zatorre et al., 1992; Jones-Gotman and Zatorre, 1993; Hummel et al., 1995). This functional lateralization of OFC may explain the underlying mechanism of the opposite effects obtained with MNS vs. auricular VNS with high frequency (80 Hz) electrostimulation. The “smell image” may also be another factor that may contribute the suppression of olfactory function with high frequency MNS in the present study. It has been reported that each odor has been coded with its integrating senses in the brain. The smell image is described as an unconscious perception, influenced by multi-sensory inputs (including vision, sound, somatosensory-touch, taste and smell), contributing to our perception of smell that arises from the sub modalities of the somatosensory system (Shepherd, 2006). In the current study, use of high frequency MNS of 80 Hz may potentially decrease the activation of S-I (as a component of smell image) as in the reports of 50 Hz MNS study (Davis et al., 1995) and this may lead to the suppression of the smell image.

In the present study, weak, moderate and strong concentrations of 4 odors (Citral, Isovaleric Acid, Amyl Acetate and 1-Octen-3-ol) have been used. The results demonstrated that high frequency (80 Hz) MNS was capable of olfactory supra-threshold suppression for moderate concentration of Amyl Acetate, strong concentration of Isovaleric Acid and strong concentration of 1-Octen-3-ol. However, this effect was not present for the remainder. Effects on different concentrations of odors *via* MNS were not completely surprising as different odorants and different concentrations of each odorant can activate different sets of olfactory receptors (ORs; Kajiya et al., 2001; Buck, 2004). This suggests that effects of MNS could be as early as the encoding stage of olfactory processing. To confirm this hypothesis, future studies could use a combination of calcium imaging and electrophysiological techniques (Touhara, 2002) or single-cell reverse transcription-polymerase chain reaction techniques (Kajiya et al., 2001) in rodent models and explore if the specific ORs that are expressed under the concentrations of odors in the current study are suppressed after high frequency MNS. NIRS recording of the OFC was performed in the current study as it has been indicated in the existing literature that presentation of olfactory stimuli is represented in this region of the cortex (Ishimaru et al., 2004; Harada et al., 2006; Kobayashi et al., 2012). However, to understand how and what part of the neural olfactory system is affected by MNS (in this particular instance, high frequency MNS), investigations of the full neural circuitry of connections that MNS propagates to before reaching the OFC is necessary. This includes the potential pathways that MNS entails to reach the olfactory regions of the cortex/sub-cortex that is indicated in Figure 5. This could be performed in future investigations using magneto encephalography or fMRI, observing the neurocircuitry that is associated with MNS and specific olfactory tests. In particular, it would help address why high frequency MNS only had an effect on specific concentrations of odors in the present study.

The networks that relays the effects of MNS to the olfactory networks is potentially acting through the vagal brainstem nuclei. NTS is a recipient of direct neural inputs from the afferent (sensory) vagus as well as the direct and indirect inputs from the pharyngeal, glossopharyngeal and trigeminal nerves, the spinal tract, the area postrema, the hypothalamus, the cerebellum and vestibular/labyrinthine systems as well as the cerebral cortex, all of which play important roles in regulation of medullary reflexes controlling nausea and vomiting (Babic and Browning, 2014). These shared effects by all corresponding nerves is thought to intersect at the NTS, which is considered a focal point of neuroanatomical intersection center for pathways associated with the peripheral and cranial nerves of the scalp, face, auricular and body (van der Kooy et al., 1984; Ruggiero et al., 2000; Cakmak, 2006). MNS relays to the NTS (Noguchi and Hayashi, 1996; Zhang et al., 2002; Tada et al., 2003; Guan and Wu, 2005; Cakmak, 2006; Imai et al., 2008; Wang et al., 2015) where afferents for locus coeruleus (LC; Chandler et al., 2014) and raphe nucleus (RN) are present (Sawchenko, 1983; Ruggiero et al., 2000; Mello-Carpes and Izquierdo, 2013; Frangos et al., 2015). LC and RN, can innervate the olfactory tubercle (OT) using noradrenergic and serotonergic fibers respectively (Solano-Flores et al., 1980; Guevara-Guzman et al., 1991; Wesson and Wilson, 2011). Both of these structures can project to the OFC, directly or through the OT (Kannan and Yamashita, 1985; Mooney et al., 1987; Ikemoto, 2007; Price, 2010; Wesson and Wilson, 2011; Chandler et al., 2014; Zhou et al., 2015). LC and RN can also be stimulated through an indirect stimulation through the NTS using MNS (Figure 5). In addition, MNS could also follow the pathway from the NTS to the insula, which can activate the primary olfactory centers (piriform cortex-PC and entorhinal cortex-EC) and OFC (Mayer, 2011). Therefore, MNS could influence the OT, OFC and OB, *via* the LC, RN and insula (Figure 5).

In addition to the neuroanatomical insights, findings from the current study also give practical supports to use MNS as a way to ameliorate nausea symptoms in patients. Drawing evidence from interviews with cancer patients, it has been identified that some strong smells (e.g., shaving cream) and food-related smells (e.g., cooked meat, frying food) can trigger or aggravate nauseas, which aversively impact on patients’ food intake (McGreevy et al., 2014; Olver et al., 2014). The current study found that MNS can effectively suppress the intensity perception of three of the four food-related odor compounds. Notably, two of these odorants are generally described by the population as unpleasant (i.e., cheese, mushroom; Ventanas et al., 2010; Mcrae et al., 2013). It is known from the previous literature that OFC is not only responsible for sensory integration, but also for assigning reward values to sensory stimulus (Rolls et al., 2003). The current finding indicates that the unpleasant odorants may be more susceptible to the modulatory effect of MNS. Although more research is needed to confirm whether MNS has differentiating effects on pleasant and unpleasant odor groups, its noticeable effects on Isovaleric Acid and 1-Octen-3-ol are promising for future investigations in the use of MNS on suppressing nausea-triggering olfactory perceptions. This non-invasive approach can particularly help cancer patients who commonly experience the cluster of symptoms associated with nausea, vomiting and loss of appetite (Guerdoux-Ninot et al., 2016).

The current study has some limitations. In the present study, only acute effects of high- and low frequency MNS were tested and chronic effects of MNS should also be investigated in future studies. The LMS test was selected for testing supra-threshold intensity perception due to its common applications in studying chemical senses. However, the LMS test procedure did not allow to observe odor-specific responses due to the presentation of stimuli without rigorous time controls. Future studies should replicate the current experimental design with different supra-threshold olfactory tests and with the use of a more accurate odor delivery system (such as a neuroimaging-compatible olfactometer). In addition, the use of NIRS techniques in the current experiment measured only the OFC (VWrSO_2_%). fMRI or magneto encephalography approaches would be useful to clarify the modulation of olfactory networks by MNS. It should also be reiterated that the current study only included healthy male participants. Female participants were excluded from the study because olfactory functioning fluctuates across menstrual cycles, which could introduce biases with the current repeated-measures design. Building on the positive findings from the current study, future studies can examine effects on female cohorts incorporating calibration of menstrual cycles in the experimental design. The last but not the least, the significant results were obtained with the conservative approach of Bonferroni correction in the present study and we also provided the effect size analysis of significant results for future meta-analysis. The effect size analysis indicated low effect size for the significant LMS results and large effect size for the significant NIRS results. It’s worth to note that low effect size for significant LMS results which are correlated with large effect size in NIRS (cortical data) do not clarify or indicate a weak or strong behavioral effect of MNS in the present study design. The clinical significance or clinical usability of MNS should be investigated in future studies with behavioral designs.

In conclusion, the present research investigated the potential role of MNS using high and low frequencies on olfactory intensity perception in healthy, adult, male participants, with supplementary exploration of the OFC. The present study indicated for the first time in human research that non-invasive high frequency MNS is able to modulate human olfactory functioning, accompanied by observation of increased activation in bilateral hemispheres of the OFC. Future studies should explore effects of MNS with high- and low frequencies on separate olfactory functioning, such as odor recognition, odor memory and odor identification, in combination with functional neuroimaging techniques, in order to understand the modulatory effect of MNS on specific olfactory-related neural circuitries.

## Author Contributions

YC: concept idea. YC and MP: project design. AM, YC and MP: performing experiments and data collection statistical analysis and preparation of final manuscript. MP and AM: interpretation of the results. YC and AM: draft manuscript.

## Conflict of Interest Statement

The authors declare that the research was conducted in the absence of any commercial or financial relationships that could be construed as a potential conflict of interest.
