# Supplementary material for: Non-invasive High Frequency Median Nerve Stimulation Effectively Suppresses Olfactory Intensity Perception in Healthy Males
Source: Front Hum Neurosci. 2019 Jan 21;12:533. doi: 10.3389/fnhum.2018.00533 (PMC6348262; doi:10.3389/fnhum.2018.00533)
Supplement: Supplementary file 1 [file Table_1.docx]

**Supplementary Table S1: Bench top testing for LMS odor selection**

Four phases of Bench top testing were performed to find three levels of concentrations for each odor that could be differentiated into weak, moderate and strong concentrations. Each testing phase has a total n of 6-10 participants per testing phase. The ratings of the LMS scale was used from the software ‘Compusense’ (Compusense Cloud, Version 8.8.6766.17069, Compusense Inc, Ontario, Canada), which has a scoring system of 0-1.980 (0.000 -no sensation, 0.140-barely detectable, 0.760-weak, 1.210-moderate, 1.520-strong, 1.700-very strong, 1.980-Strongest imaginable). This scale was later adapted using a line scale for the current study using a 0-100 scale following protocols of (Green et al., 1996, 1993; Kalva et al., 2014).

**Bench top testing 1**

| **Odor** | **Mean** | **Standard Deviation** | **P-value** |
| --- | --- | --- | --- |
| **Citral** | | | |
| 10ppm | 0.815 | 0.419 | 0.001 |
| 50ppm | 1.426 | 0.155 |  |
| 100ppm | 1.45 | 0.257 |  |
| **Amyl Acetate** | | | |
| 10ppm | 0.909 | 0.382 | 0.0001 |
| 50ppm | 1.352 | 0.256 |  |
| 100ppm | 1.259 | 0.227 |  |
| **Isovaleric Acid** |  |  |  |
| 10ppm | 1.103 | 0.376 | 0.004 |
| 50ppm | 1.351 | 0.289 |  |
| 100ppm | 1.244 | 0.282 |  |
| **1-Octen-3-ol** |  |  |  |
| 10ppm | 0.496 | 0.483 | 0.0001 |
| 50ppm | 1.409 | 0.176 |  |
| 100ppm | 1.301 | 0.249 |  |
| **Ethyl Butyrate** |  |  |  |
| 10ppm | 0.931 | 0.336 | 0.0001 |
| 50ppm | 1.369 | 0.303 |  |
| 100ppm | 1.375 | 0.246 |  |

**Bench top testing 2**

| **Odor** | **Mean** | **Standard Deviation** | **P-value** |
| --- | --- | --- | --- |
| **Citral** | | | |
| 2ppm | 0.731 | 0.361 | 0.001 |
| 20ppm | 1.332 | 0.109 |  |
| 200ppm | 1.634 | 0.111 |  |
| **Amyl Acetate** | | | |
| 20ppm | 1.03 | 0.344 | 0.018 |
| 12ppm | 1.322 | 0.253 |  |
| 70ppm | 1.505 | 0.229 |  |
| **Isovaleric Acid** |  |  |  |
| 2ppm | 0.895 | 0.213 | 0.033 |
| 12ppm | 0.996 | 0.457 |  |
| 70ppm | 1.266 | 0.192 |  |
| **1-Octen-3-ol** |  |  |  |
| 10ppm | 1.339 | 0.25 | 0.06 |
| 23ppm | 1.236 | 0.279 |  |
| 50ppm | 1.568 | 0.127 |  |
| **Ethyl Butyrate** |  |  |  |
| 2ppm | 1.078 | 0.21 | 0.0001 |
| 17ppm | 1.536 | 0.134 |  |
| 150ppm | 1.555 | 0.139 |  |

**Bench top testing 3**

| **Odor** | **Mean** | **Standard Deviation** | **P-value** |
| --- | --- | --- | --- |
| **Citral** | | | |
| 2ppm | 1.4 | 0.179 | 0.083 |
| 20ppm | 1.473 | 0.281 |  |
| 200ppm | 1.666 | 0.111 |  |
| **Amyl Acetate** | | | |
| 1.25ppm | 0.748 | 0.532 | 0.009 |
| 10ppm | 0.949 | 0.359 |  |
| 80ppm | 1.49 | 0.141 |  |
| **Isovaleric Acid** |  |  |  |
| 5ppm | 0.895 | 0.204 | 0.002 |
| 27.39ppm | 1.274 | 0.179 |  |
| 150ppm | 1.399 | 0.22 |  |
| **1-Octen-3-ol** |  |  |  |
| 5ppm | 1.046 | 0.36 | 0.005 |
| 20ppm | 1.26 | 0.276 |  |
| 80ppm | 1.565 | 0.171 |  |
| **Ethyl Butyrate** |  |  |  |
| 2ppm | 1.225 | 0.321 | 0.041 |
| 20ppm | 1.301 | 0.231 |  |
| 200ppm | 1.49 | 0.118 |  |

**Bench top testing 4**

| **Odor** | **Mean** | **Standard Deviation** | **P-value** |
| --- | --- | --- | --- |
| **Citral** | | | |
| 2ppm | 0.5812 | 0.3769 | 0.0001 |
| 20ppm | 1.3163 | 0.2522 |  |
| 200ppm | 1.3923 | 0.2958 |  |
| **Amyl Acetate** | | | |
| 1.5ppm | 0.5095 | 0.3897 | 0.001 |
| 15ppm | 1.1107 | 0.2923 |  |
| 150ppm | 1.517 | 0.1895 |  |
| **Isovaleric Acid** |  |  |  |
| 4ppm | 0.917 | 0.442 | 0.008 |
| 28ppm | 1.3002 | 0.2591 |  |
| 196ppm | 1.4835 | 0.2484 |  |
| **1-Octen-3-ol** |  |  |  |
| 3ppm | 0.9375 | 0.5385 | 0.012 |
| 18ppm | 1.1363 | 0.2757 |  |
| 108ppm | 1.5392 | 0.1706 |  |
| **Ethyl Butyrate** |  |  |  |
| 1.25ppm | 0.914 | 0.358 | 0.003 |
| 13.69ppm | 1.216 | 0.305 |  |
| 150ppm | 1.582 | 0.2344 |  |
