# Supplementary material for: Non-invasive High Frequency Median Nerve Stimulation Effectively Suppresses Olfactory Intensity Perception in Healthy Males
Source: Front Hum Neurosci. 2019 Jan 21;12:533. doi: 10.3389/fnhum.2018.00533 (PMC6348262; doi:10.3389/fnhum.2018.00533)
Supplement: Supplementary file 2 [file Table_2.docx]

**Supplementary Table S2: Reference list that corresponds to citations in Figure 5**

1. Songzi W, Lu R, Lianqun J, Zhongyue G, Xu F, Wenna C, et al. Effect of acupuncture at Neiguan ( PC 6 ) on cardiac function using echocardiography in myocardial ischemia rats induced by isoproter-enol. J Tradit Chinese Med [Internet]. 2015;35(6):653–8. Available from: <http://dx.doi.org/10.1016/S0254-6272(15)30155-2>
2. Tada H, Fujita M, Harris M, Tatewaki M. Neural Mechanism of Acupuncture-Induced Gastric Relaxations in Rats. Dig Dis Sci. 2003;48(1):59–68.
3. Imai K, Ariga H, Chen C, Mantyh C, Pappas TN, Takahashi T. Effects of electroacupuncture on gastric motility and heart rate variability in conscious rats. Auton Neurosci Basic Clin. 2008;138(1–2):91–8.
4. Noguchi E, Hayashi H. Increases in gastric acidity in response to EA stimulation of hindlimb of anesthetized rats. Jpn J Physiol. 1996;46:53–8.
5. Cakmak YO. Epilepsy, electroacupuncture and the nucleus of the solitary tract. Acupunct Med. 2006;24(4):164–8.
6. Zhang G, Yin H, Zhou Y-L, Han H-Y, Wu Y-H, Xing W, et al. Capturing Amplitude Changes of Low-Frequency Fluctuations in Functional Magnetic Resonance Imaging Signal : A Pilot Acupuncture Study on NeiGuan (PC6). J Altern Complement Med. 2012;18(4):387–93.
7. Guan YH, Wu XP. The influence of electroacupunturing Neiguan on the content of ET, TXB2, and 6-Keto-PGF1a in acute myocardial ischemia rabbits. Hu Bei Zhong Yi Xue Yuan Xue Bao. 2005;7(2):13–4.
8. Tatewaki M, Strickland C, Fukuda H, Tsuchida D, Hoshino E, Pappas TN, et al. Effects of acupuncture on vasopressin-induced emesis in conscious dogs. Am J Physiol Integr Comp Physiol. 2005;288(2):401–8.
9. van der Kooy D, Koda LY, McGinty JF, Gerfen CR, Bloom FE. The organization of projections from the cortex, amygdala, and hypothalamus to the nucleus of the solitary tract in rat. J Comp Neurol [Internet]. 1984;224(1):1–24. Available from: <http://www.ncbi.nlm.nih.gov/pubmed/6715573>
10. Li J, Li J, Chen Z, Liang F, Wu S, Wang H. The influence of PC6 on cardiovascular disorders: a review of central neural mechanisms. Acupunct Med. 2012;30(1):47–50.
11. Frangos E, Ellrich J, Komisaruk BR. Non-invasive access to the vagus nerve central projections via electrical stimulation of the external ear: FMRI evidence in humans. Brain Stimul [Internet]. 2015;8(3):624–36. Available from: <http://dx.doi.org/10.1016/j.brs.2014.11.018>
12. Ruggiero DA, Underwood MD, Mann JJ, Anwar M, Arango V. The human nucleus of the solitary tract: visceral pathways revealed with an “in vitro” postmortem tracing method. J Auton Nerv Syst. 2000;79:181–90.
13. Sawchenko PE. Central connections of the sensory and motor nuclei of the vagus nerve. J Auton Nerv Syst. 1983;9:13–26.
14. Mello-Carpes PB, Izquierdo I. The nucleus of the solitary tract→nucleus paragigantocellularis→locus coeruleus→CA1 region of dorsal hippocampus pathway is important for consolidation of object recognition memory. Neurobiol Learn Mem. 2013;100:56–63.
15. Chandler DJ, Gao W-J, Waterhouse BD. Heterogeneous organization of the locus coeruleus projections to prefrontal and motor cortices. Proc Natl Acad Sci [Internet]. 2014;111(18):6816–21. Available from: <http://www.pnas.org/cgi/doi/10.1073/pnas.1320827111>
16. Wesson DW, Wilson DA. Sniffing out the contributions of the olfactory tubercle to the sense of smell: hedonics, sensory integration, and more? Neurosci Biobehav Rev. 2011;35:655–68.
17. Solano-Flores LP, Aguilar-Baturoni HU, Guevara-Aguilar R. Locus coeruleus influences upon the olfactory tubercle. Brain Res Bull. 1980;5:383–9.
18. Guevara-Guzman R, García-Díaz DE, Solano-Flores LP, Wayner MJ, Armstrong DL. Role of the paraventricular nucleus in the projection from the nucleus of the solitary tract to the olfactory bulb. Brain Res Bull. 1991;27(3):447–50.
19. Mooney KE, Inokuchi A, Snow Jr JB, Kimmelman CP. Projections from the ventral tegmental area to the olfactory tubercle in the rat. Otolaryngol Neck Surg. 1987;96:151–7.
20. Kannan H, Yamashita H. Connections of neurons in the region of the nucleus tractus solitarius with the hypothalamic paraventricular nucleus: Their possible involvement in neural control of the cardiovascular system in rats. Brain Res. 1985;329(1–2):205–12.
21. Ikemoto S. Dopamine reward circuitry: two projection systems from the ventral midbrain to the nucleus accumbens-olfactory tubercle complex. Brain Res Rev. 2007;56(1):27–78.
22. Price JL. Olfactory Higher Centers Anatomy. 2010. 129-136 p.
23. Zhou J, Jia C, Feng Q, Bao J, Luo M. Prospective coding of dorsal raphe reward signals by the orbitofrontal cortex. J Neurosci. 2015;35:2717–30.
24. Mayer EA. Gut feelings: the emerging biology of gut–brain communication. Nat Rev Neurosci. 2011;12(8):1–30.
25. Ruggiero DA, Anwar S, Kim J, Glickstein SB. Visceral afferent pathways to the thalamus and olfactory tubercle: Behavioral Implications. Brain Res. 1998;799(1):159–71.
26. Powell BYTPS, Cowan WM, Raisman G. The central olfactory connexions. J Anat. 1965;99(4):791–813.
27. Savic I, Gulyas B, Larsson M, Roland P. Olfactory Functions Are Mediated by Parallel and Hierarchical Processing. Neuron. 2000;26:735–45.
28. Shute CCD, Lewis PR. The ascending cholinergic reticular system: neocortical, olfactory and subcortical projections. Brain. 1967;90(3):497–520.
29. Gervais R. Unilateral lesions of the olfactory tubercle modifying general arousal effects in the rat olfactory bulb. Electroencephalogr Clin Neurophysiol. 1979;46(6):665–74.
30. Chen JDZ, Qian L, Ouyang H, Yin J. Gastric electrical stimulation with short pulses reduces vomiting but not dysrhythmias in dogs. Gastroenterology. 2003;124(2):401–9.
31. Ouyang HUI, Yin J, Wang Z, Pasricha PJ, Chen JDZ, Yin J, et al. Electroacupuncture accelerates gastric emptying in association with changes in vagal activity. Am J Gastrointest Liver Physiol. 2002;282:390–6.
32. Berthoud HR, Neuhuber WL. Functional and chemical anatomy of the afferent vagal system. Auton Neurosci Basic Clin. 2000;85(1–3):1–17.
33. Xu S, Hou X, Zha H, Gao Z, Chen Y, Zhang JDZ. Electroacupuncture Accelerates Solid Gastric Emptying and Improves Dyspeptic Symptoms in Patients with Functional Dyspepsia. Dig Dis Sci. 2006;51(12):2154–9.
34. Takahashi T. Mechanism of Acupuncture on Neuromodulation in the Gut — A Review. Neuromodulation. 2011;14:8–12.
35. Dhond RP, Yeh C, Park K, Kettner N, Napadow V. Acupuncture modulates resting state connectivity in default and sensorimotor brain networks. Pain. 2008;136(3):407–18.
36. Ren Y, Bai L, Feng Y, Tian J, Li K. Investigation of acupoint specificity by functional connectivity analysis based on graph theory. Neurosci Lett. 2010;482:95–100.
37. Yang H, Wu S, Zheng Q, Xiao C, Liu X, Xiao Y, et al. Meta-analysis on protective effect of electroacupuncture at “Neiguan” (PC 6) in rats with reperfusion injury induced myocardial ischemia. World J Acupuncture-Moxibustion [Internet]. 2015;25(3):43–53. Available from: <http://dx.doi.org/10.1016/S1003-5257(15)30063-5>
38. Travagli RA, Hermann GE, Browning KN, Rogers RC. NIH Public Access. Annu Rev Physio. 2011;68(7):279–305.
39. Hayakawa T, Takanga A, Tanaka K, Maeda S, Seki M. Organization and distribution of the upper and lower esophageal motoneurons in the medulla and the spinal cord of the rat. Okajimas Folia Anat Jpn. 2002;78(6):263–79.
40. Norgren R, Smith GP. Central distribution of subdiaphragmatic vagal branches in the rat. J Comp Neurol. 1988;273(2):207–23.
