# Supplementary figures and images for: Non-invasive High Frequency Median Nerve Stimulation Effectively Suppresses Olfactory Intensity Perception in Healthy Males
Source: Front Hum Neurosci. 2019 Jan 21;12:533. doi: 10.3389/fnhum.2018.00533 (PMC6348262; doi:10.3389/fnhum.2018.00533)

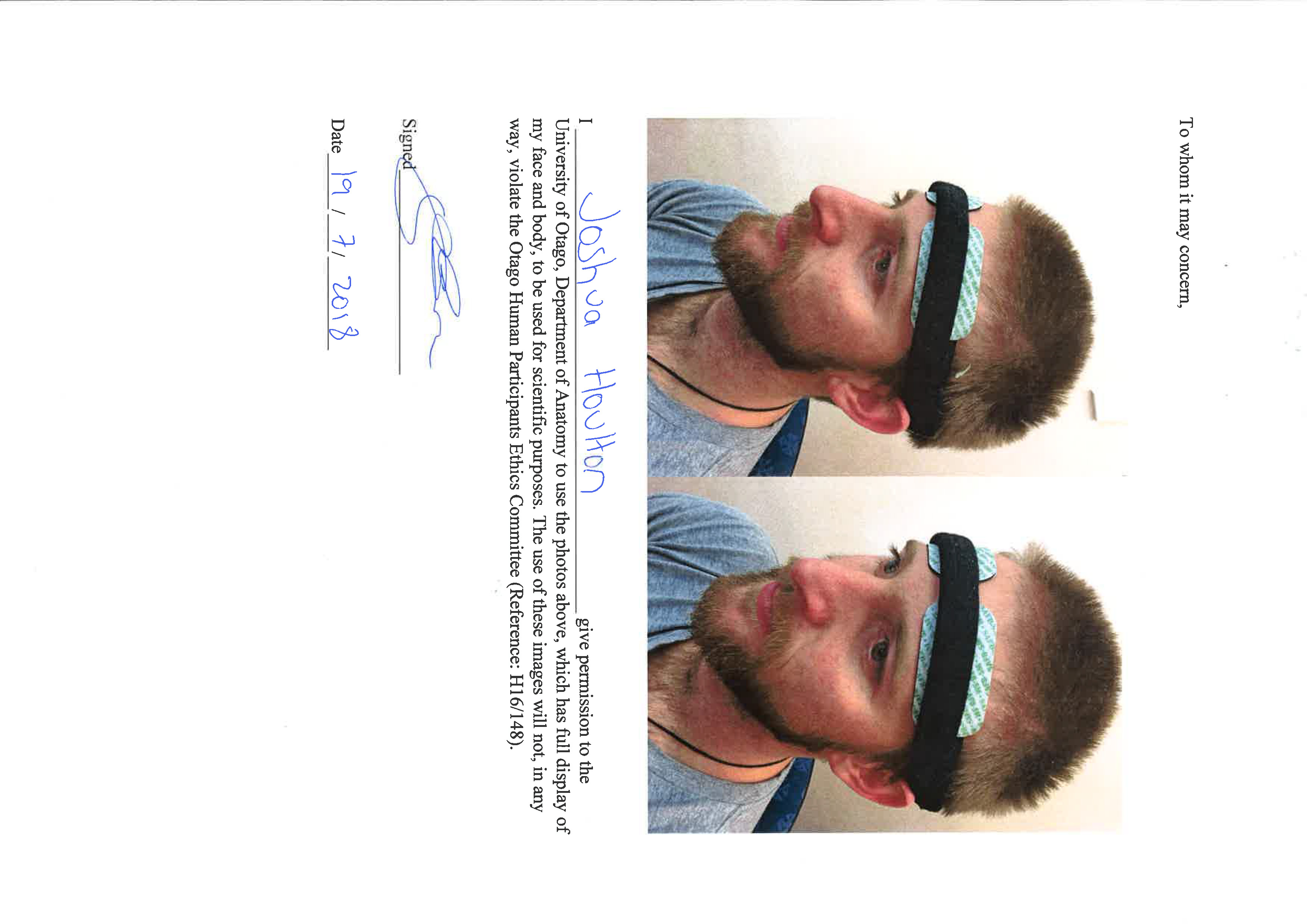

Supplement: Supplementary file 3 [file Image_1.JPEG]
